# Sustained real-time and video-rate interventional optical ultrasound imaging

**DOI:** 10.1117/1.JBO.30.3.036005

**Published:** 2025-03-21

**Authors:** Robert M. Stafford-Williams, Richard J. Colchester, Semyon Bodian, Seán Cardiff, Efthymios Maneas, Edward Z. Zhang, Paul C. Beard, Manish K. Tiwari, Adrien E. Desjardins, Erwin J. Alles

**Affiliations:** a University College London, Department of Medical Physics & Biomedical Engineering, Faculty of Engineering Sciences, London, United Kingdom; b University College London, Wellcome/EPSRC Centre for Interventional and Surgical Sciences, Faculty of Engineering Sciences, London, United Kingdom; c University College London, UCL Mechanical Engineering, Faculty of Engineering Sciences, London, United Kingdom

**Keywords:** interventional optical ultrasound imaging, linearly actuated aperture scanning, oblique optical ultrasound emitter, pulmonary optical ultrasound

## Abstract

**Significance:**

Minimally invasive surgery offers improved recovery times and reduced complication risk compared with open surgery. However, effective image acquisition probes suitable for deployment in clinical workflows are key to the success of such procedures. Fiber-optic optical ultrasound (OpUS) offers strong potential for interventional image guidance due to its small lateral probe dimensions and high imaging resolution, but to date, such miniature imaging probes have only yielded M-mode (single image line) or still images.

**Aim:**

Here, we present a motorized actuation approach to fiber-optic interventional OpUS imaging that enables sustained and video-rate imaging while retaining its small form factor.

**Approach:**

A fabrication method utilizing a commercial laser cutter is presented that yields partially forward-emitting OpUS sources ideally suited for interventional image guidance. These transmitters were incorporated into a miniature imaging probe with a width of just 600  μm (1.8 mm with protective encapsulation) and combined with a linear actuator to synthesize an imaging aperture at the distal end of the probe through manipulation at its proximal end.

**Results:**

The presented imaging paradigm achieved real-time, two-dimensional OpUS imaging at frame rates of up to 7 Hz and was capable of high-resolution imaging (94  μm axial and 241  μm lateral). The imaging performance of the presented imaging system was assessed using various imaging phantoms, and its clinical suitability was confirmed by emulating endobronchial OpUS imaging through a commercial bronchoscope.

**Conclusions:**

These results constitute the first-ever sustained, real-time dynamic imaging using a side-viewing single-element OpUS probe via rapid actuation, which enables a wide range of applications in minimally invasive surgical guidance.

## 1 Introduction

Minimally invasive or interventional surgery requires high-quality image guidance to be successful. Although predominantly performed under guidance from externally applied modalities [e.g., magnetic resonance imaging (MRI), X-ray computed tomography (CT), and ultrasound], such external modalities suffer from significant drawbacks:^1^ CT imaging delivers ionizing radiation and yields limited soft-tissue contrast, MRI requires intense magnetic fields that preclude certain patients and most surgical instruments and typically does not offer fast or real-time imaging, and externally applied ultrasound imaging offers limited spatial resolution.

Interventional image guidance is better suited to guide minimally invasive surgery. Performed from within surgical instruments directly at the surgical site, such image guidance offers the highest possible spatio-temporal resolution and contrast without significantly interfering with the procedure. However, current interventional imaging modalities offer limited performance. For instance, endoscopy and optical coherence tomography offer limited depth penetration,^2^ endobronchial ultrasound (EBUS) or intracardiac echocardiography probes are too large to reach all clinically relevant anatomical features,^3^ and intravascular ultrasound (IVUS) or radial EBUS generate circumferential images oriented orthogonal to the imaging probe long axis^4^ that—while offering diagnostic value—are of limited use for guiding instrument navigation.

Recently, optical ultrasound (OpUS) imaging has been demonstrated as a viable alternative to conventional electronic ultrasound technology. With OpUS imaging, pulsed excitation light is delivered to an optically absorbing structure, where it is converted via the photoacoustic effect^5^ into ultrasound waves that propagate through the tissue and reflect off tissue interfaces. These back-scattered waves in turn impinge on microscopic optically resonant structures such as Fabry–Pérot cavities, ring resonators, or Bragg gratings,^6^ resulting in temporal modulation of their resonance condition that can be monitored using a photodiode. The broadband and high-amplitude OpUS generation,^7^ combined with exquisitely sensitive OpUS detection, result in high-resolution, high-quality images and excellent soft tissue contrast at depths of up to several centimeters.^8^ Using off-the-shelf fiber-optic components that do not require elaborate electromagnetic shielding, OpUS imaging probes are readily miniaturized and potentially cost-effective. Such fiber-optic probes have been demonstrated to achieve high-quality two-dimensional (2D) and three-dimensional (3D) images of *ex vivo* tissue samples^9^^,^^10^ through slow motorized raster scanning, both *in vivo*^11^ and *ex vivo* M-mode imaging^8^ to monitor and inform surgical procedures using just a single image line, and visualization of *ex vivo* tissue through a rapid, single pull-back of the imaging probe^12^ that offers diagnostic potential but is of limited value for image guidance. To date, “video-rate,” sustained OpUS imaging (as opposed to single-frame or short burst acquisition) has only been achieved using externally applied hand-held probes^13^^,^^14^ or clinically impractical bench-top systems.^15^^,^^16^

Here, a novel OpUS imaging platform presented that, for the first time, achieves sustained, real-time, and video-rate OpUS imaging, using a miniature fiber-optic probe well-suited to interventional use. This is achieved through rapid and repeated linear actuation of an imaging probe at its proximal end, resulting in periodic rapid scanning of a synthetic imaging aperture at its distal end. As fiber-optics offer both stiffness (to translate the actuator motion to the probe tip) and flexibility (to allow for versatile, interventional deployment) in a highly compact form factor, this approach results in highly controlled probe motion and manipulation and corresponding high image quality. Using optical fiber processing techniques, OpUS imaging probes are presented that emit ultrasound obliquely, thus generating a partially forward-facing field of view—which is highly preferred for image guidance during instrument placement—yet feature lateral dimensions similar or smaller than those of IVUS and radial EBUS probes.

This paper describes the fabrication, actuation, and data acquisition processes of this linearly actuated OpUS (LiOpUS) platform. In addition, acoustic measurements and phantoms were used to assess its imaging performance. Finally, the first application of LiOpUS to interventional pulmonary imaging was demonstrated on a tissue-mimicking lung phantom.

## 2 Methods

### 2.1 LiOpUS Imaging Paradigm

**Fig. 1:**
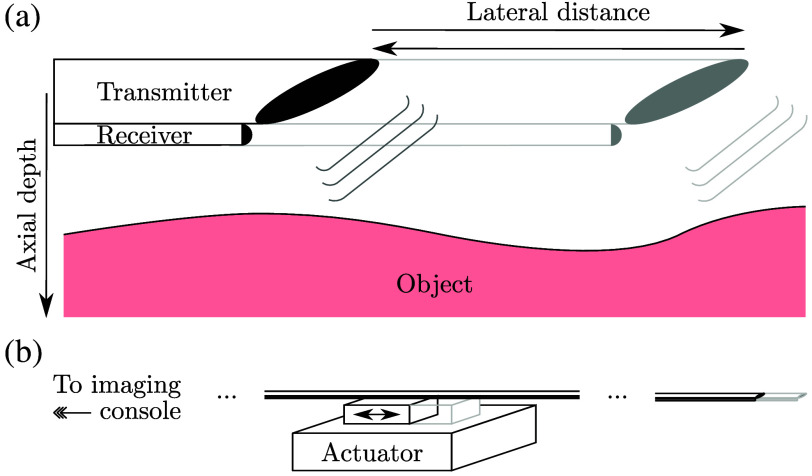
LiOpUS imaging paradigm. (a) A fiber-optic optical ultrasound imaging probe is rapidly actuated along its long axis to repeatedly scan and synthesize a linear aperture located at its distal end while continuously acquiring pulse-echo signals. Ultrasound is emitted obliquely to obtain a 2D imaging plane that is oriented parallel to the direction of motion and is partially forward-facing. The lateral (horizontal) and axial (vertical) directions are defined as indicated. (b) Rapid distal aperture scanning is achieved by mounting the proximal end of the fiber-optic probe to a linear voice coil actuator programmed to traverse its travel range in a back-and-forth fashion. This motion is accurately translated to the distal end through the stiffness of the optical fibers.

To achieve video-rate aperture scanning, a fiber-optic OpUS probe was mounted proximally to a linear voice coil actuator capable of periodically traversing an aperture width of 12 mm at up to 25  cycles/s (Fig. 1). Exploiting the stiffness of its constituent optical fibers and spatial confinement of its protective sheath, this imaging probe (measuring ca. 1.5 m in length) accurately transferred the motion of the proximal actuator to distally scan an imaging aperture. A custom, laser-cut assembly was used to achieve firm mounting while allowing for rotational manipulation.

### 2.2 Imaging Probe Fabrication

Contrary to previous work, where ultrasound was emitted in either forward-^9^^,^^17^^–^^21^ or side-viewing configuration,^12^^,^^22^^,^^23^ the OpUS imaging probe used in this work was designed to emit ultrasound obliquely. Forward-facing imaging probes are of limited value in interventional applications as spatial constraints preclude the lateral actuation required to synthesize an imaging aperture, and purely side-viewing probes—while yielding clinically relevant images—are of limited value during instrument navigation which requires visualization of the area ahead of the instrument.

**Fig. 2:**
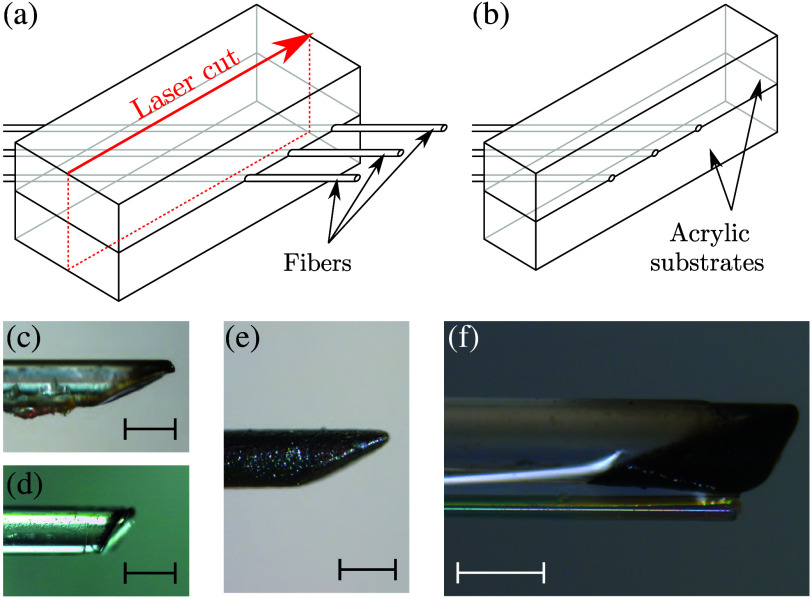
Fabrication of obliquely emitting fiber-optic ultrasound sources. (a) Multi-mode optical fibers are placed in angled grooves laser-engraved in acrylic substrates and bonded in place with a temporary adhesive. (b) This assembly is laser-cleaved to obtain a batch of optical fibers cleaved at reproducible and well-controlled angles. (c) and (d) Microscope images of fibers cleaved at 60 deg (c) and 45 deg (d). (e) Microscope image of a 60-deg cleaved optical fiber after deposition of the photoacoustic generating coating. (f) Microscope image of a fully assembled fiber-optic imaging probe comprising a 45-deg cleaved source (top) and fiber-optic Fabry–Pérot ultrasound detector (bottom). Scale bars: 400  μm.

To achieve oblique OpUS emission, a novel and facile two-step source fabrication method was developed that allowed for flexible emission angles and a monolithic structure (as opposed to, for example, labor-intensive capillary assemblies^22^ requiring accurate alignment and placement). To this aim, a commercial laser cutter was used to angle-cleave large-core multimode optical fibers to the desired emission angle, and the resulting cleaved surface was coated in a photoacoustic generating material (Fig. 2). Specifically, optical fibers were sandwiched between acrylic substrates (thickness: 3 mm each), which contained angled, engraved grooves to facilitate alignment. The fibers and substrates were temporarily bonded using a UV-curable blocking adhesive (NBA 107, Norland Products, Jamesburg, New Jersey, United States) and laser-cut (power: 100% and speed: 2.5%; VLS 4.60, Universal Laser Systems, Scottsdale, Arizona, United States) using a trajectory parallel to the substrate edge [Figs. 2(a) and 2(b)]. A heat gun was then applied to remove the blocking adhesive, and any remaining excess adhesive was removed manually with a scalpel [Figs. 2(c) and 2(d)].

The tips of these fibers were subsequently coated with a mixture of a carbon black optical absorber in a polydimethylsiloxane (PDMS) elastomeric host.^24^ This mixture was prepared by first mixing carbon black (SKU 699632, Sigma-Aldrich, St. Louis, Missouri, United States) into a Neo-Clear solvent (SKU 1098435000, Sigma-Aldrich, St. Louis, Missouri, United States) using a planetary mixer (SpeedMixer DAC 330-100 SE, FlackTek, Landrum, South Carolina, United States) to obtain a 5% wt/wt solution. This solution was subsequently mixed into PDMS (MED-1000, NuSil Technology, Carpinteria, California, United States; 2 ml solution per 1 g PDMS) and finally applied to the angle-cleaved sources through manual dip-coating followed by a 24-h cure at ambient conditions. The resulting coating [Fig. 2(e)] efficiently converted pulsed excitation light into broadband ultrasound via the photoacoustic effect.^5^

This fabrication process resulted in fiber-optic OpUS sources with an angled, elliptical surface, where the cleave angle determined both the emission direction and spatial divergence of the ultrasound.^25^ In this work, a fiber with a core diameter of 400  μm (FT400UMT, Thorlabs, Dortmund, Germany) was chosen as a compromise among mechanical robustness, small lateral size, and predicted acoustical performance, which was cleaved at angles of either 45 or 60 deg to demonstrate the versatility of the fabrication method.

The resulting OpUS source fiber was paired with a custom plano-concave fiber-optic ultrasound detector,^10^ and the tip of which was placed at the apex of the source surface [Fig. 2(f)] and affixed using a UV-curable adhesive (NOA68, Norland Products, Jamesburg, New Jersey, United States). This detector exhibited a broadband and near-omnidirectional response.^10^ The assembled OpUS imaging probe (lateral dimension: 600  μm) was inserted into a commercially available protective sheath (outer diameter: 1.8 mm; K-201 single-use guide sheath, Olympus, Tokyo, Japan) to facilitate handling and probe positioning.

### 2.3 Acoustical Characterization

Acoustical field scans (grid size: 4  mm×4  mm, 50  μm step size; MTS25-Z8, Thorlabs, Dortmund, Germany) were performed using a calibrated needle hydrophone (diameter: 75  μm, calibration range: 1 to 30 MHz; NH0075-SYSTEM, Precision Acoustics, Dorchester, United Kingdom) and high-speed digitizer (M4i.4420-x8, Spectrum Instrumentation, Großhansdorf, Germany). The field scan plane was placed parallel to the OpUS emitting surface at a distance of 1.5 mm. This enabled quantification of the emitted bandwidth and pressure at the measurement plane; numerical propagation using the angular spectrum approach (ASA)^26^ was performed to extract full-width-at-half-maximum (FWHM) beam width metrics of the acoustic beam at various distances.

### 2.4 Image Formation and Signal Processing

**Fig. 3:**
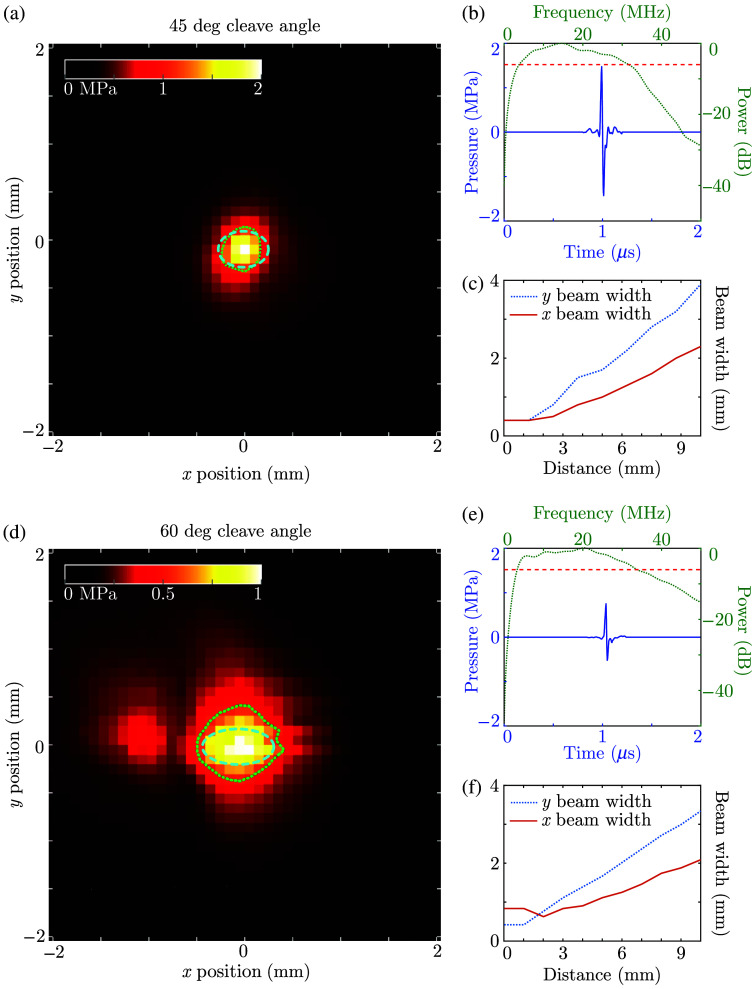
Acoustical characterization of two fiber-optic ultrasound sources. (a)–(c) Measurements of a 45-deg cleaved fiber-optic source. (a) Maximum pressure observed across a field scan measured at a distance of 1.5 mm from the source surface, with the angled source surface positioned parallel to the measurement plane, and the *x* and *y* axes oriented along the long and short axis of the source surface, respectively. The dotted green curve corresponds to the full-width half-maximum of the pressure field and is indicative of the beam width; the dashed cyan curve indicates the location and dimensions of the source surface. (b) Time trace (blue solid) and power spectrum (green dotted) extracted at the position for which the peak acoustic amplitude was observed. The red dashed line indicates the −6-dB power level used to measure the emitted bandwidth. (c) Beam widths measured along the *x* (red solid) and *y* (blue dotted) directions obtained at varying distances from the source surface through numerical propagation of the measurement plane. (d)–(f) Same presentation as in panels (a)–(c) for data obtained for a 60-deg cleaved ultrasound source.

The broad bandwidth typical for optically generated ultrasound (4 to 30 MHz, *cf.* Fig. 3) results in high source directivity for the comparatively large source dimensions (400  μm elevational, theoretically 566 or 800  μm lateral for cleave angles of 45 and 60 deg, respectively), as the source dimensions exceed the acoustic wavelength across the majority of the emitted bandwidth. As such, conventional image reconstruction algorithms such as delay-and-sum (DaS) underperform compared with when point-like sources were used. Therefore, in this work, two image formation approaches are compared.

In the first approach, the recorded pulse-echo data are band-pass filtered (4 to 50 MHz) to exclude frequencies of negligible acoustic power and hence maximize signal-to-noise ratio. The fiber-optic source element is larger than the acoustic wavelength across this entire bandwidth (≤375  μm), resulting in strong source directivity and near-collimated acoustic beams. Consecutive A-lines, which are assumed to only contain pulse-echo events occurring directly in front of the angled OpUS emitting surface, are then simply concatenated to form an image (referred to as “concatenated B-scan”) without performing a reconstruction step. This approach is potentially more robust under probe positioning uncertainty, which might be substantial given the large probe length of 1.5 m. In the second approach, a narrower band-pass filter (4 to 15 MHz) was applied to the data prior to applying a conventional DaS reconstruction algorithm. The limits of this filter were empirically found to yield a good compromise between image resolution and noise rejection. For concatenated B-scan images, the consecutive A-lines were reoriented along the emission direction to avoid image distortion and interpolated (via cubic spline interpolation) onto the same coordinate grid used for DaS-reconstructed images.

Pulse-echo signals were amplified prior to digitization (+30  dB; DHPVA-200, FEMTO, Berlin, Germany), and band-pass filtered with variable cutoff frequencies. Artifacts due to ringing within the protective sheath and direct cross-talk, where emitted ultrasound impinges onto the detector directly without scattering, were suppressed via singular value decomposition (SVD)-based filtering.^27^ Envelope detection, time-gain compensation, and log compression were applied to concatenated B-scan or DaS images to improve image visualization.

### 2.5 Experimental Setup

Pulsed excitation light (wavelength: 1064 nm, pulse repetition rate: 1 kHz, pulse duration: 1.5 ns, and pulse energy: 70  μJ; DSS1064-Q4, CryLaS, Berlin, Germany) was used to generate acoustic waves. Pulse-echo signals were detected using a custom plano-concave fiber-optic Fabry–Pérot sensor,^10^ which was interrogated using a wavelength-tunable laser (power: 15 mW and wavelength: 1500 to 1630 nm; TSL-550, Santec, Japan) tuned to the optimal bias wavelength.^28^ The reflectivity of this sensor was detected using a custom photodiode and recorded using the aforementioned digitizer.

An image frame was acquired by programming the linear voice coil actuator (XDMQ12P- DE52-KX14AG, Zaber, Vancouver, Canada) to move from 0 to 12 mm (or vice versa) at a fixed velocity while continuously acquiring pulse-echo signals and real-time actuator position readouts during motion. The pulse-echo signals (1 kHz) and positional read-outs (ca. 67 Hz) were available at different frequencies, and linear interpolation was performed to estimate the actuator position corresponding to each excitation laser pulse.

The light sources, digitizer, and actuator were controlled and synchronized by custom LabVIEW scripts running on a desktop PC (Intel Core i5-3210M quad-core CPU, 16 GB RAM, Windows 10), which achieved real-time, video-rate acquisition, signal processing, and display of concatenated B-scan data. However, SVD filtering and DaS reconstruction were performed offline only in post-processing.

### 2.6 Imaging Scenarios

The LiOpUS paradigm was demonstrated in three imaging scenarios, where LiOpUS imaging was performed using different actuator velocities—resulting in different frame rates—and using both directional concatenated B-scan and DaS-reconstructed approaches. In all three scenarios, an OpUS source cleaved at 60 deg was used. The first scenario used a phantom comprising two layers of point scatterers (tungsten wires with a diameter of 27  μm spaced 2 mm laterally and 3 mm axially, placed orthogonally to the image plane) that was used to assess the image resolution using both image formation approaches.

The second scenario used a tissue-mimicking phantom comprising gelwax,^29^ modeled after the vasculature observed on a human placenta. This phantom was of uniform construction and did not contain deliberate sub-surface features but was found to have an internal defect that upon closer inspection turned out to be a crack through the phantom. This phantom provided physiologically accurate acoustic performance but was not an accurate representation of interventional imaging.

The third scenario used an anatomically correct tissue-mimicking phantom of the human airways, containing inclusions mimicking the lymph nodes. This endobronchial phantom^30^ was fabricated from polyvinyl alcohol^31^ to achieve physiologically relevant ultrasound reflectivity and speckle levels. To more closely mimic an interventional setting, a commercial bronchoscope (11900 AP Bronchscope, KARL STORZ, Tuttlingen, Germany), which offered additional white field endoscopy, was used to deploy the LiOpUS imaging probe through the bronchoscope instrument channel.

## 3 Results

### 3.1 Imaging Probe Fabrication

Microscope images of fiber-optic OpUS sources prepared for two cleave angles (45 and 60 deg) confirmed the fabrication method enabled accurate control over the angle and shape of the emitting surface, resulted in uniform photoacoustic coating deposition, and achieved good reproducibility (Fig. 2).

### 3.2 Acoustical Performance

Both of these sources generated high pressures (peak-positive pressure: 1.4 and 0.8 MPa for 45 and 60 deg, respectively), broad bandwidths (26 and 29 MHz for 45 and 60 deg, respectively), and nearly symmetrical pressure fields (Fig. 3). However, the OpUS source cleaved at an angle of 60 deg emitted a weak secondary “beam” [visible at x=−1  mm in Fig. 3(d)], suggesting a slight inhomogeneity in the photoacoustic coating. Upon numerical propagation, these two OpUS sources were found to emit acoustic fields with similar FWHM beam widths along the source surface short axis [Figs. 3(c) and 3(f)], as was expected given their identical fiber core diameter. However, a weaker divergence with depth along the source surface long axis was observed for the source cleaved at 60 deg due to its larger surface area, in line with previous predictions.^25^

### 3.3 Resolution

**Fig. 4:**
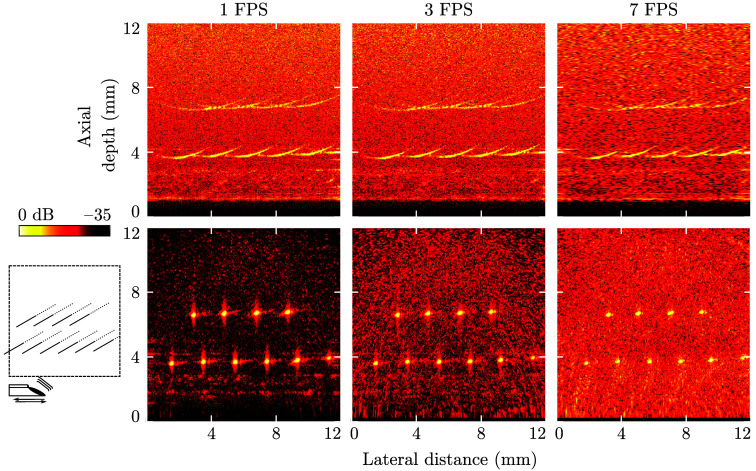
LiOpUS imaging of point targets. Images acquired at frame rates of (left to right) 1, 3, and 7 FPS. Data are presented as concatenated B-scans (top) or delay-and-sum reconstructed images (bottom). For all images, the LiOpUS probe was positioned at an axial depth of 0 mm and translated across a distance of 12 mm. The phantom consisted of tungsten wires placed orthogonal to the imaging plane, and its geometry and the location and translation direction of the LiOpUS probe are shown in the bottom-left panel.

The LiOpUS system achieved high resolutions regardless of frame rate (Fig. 4). However, a limited pulse repetition rate (PRR) of the excitation laser resulted in decreasing numbers of A-lines recorded per image frame with increasing frame rate. Consequently, the image contrast decreased with increasing frame rate. This was most significant where DaS-reconstruction was applied, as this algorithm is most accurate when high numbers of A-lines are available. For DaS-reconstructed images, the lateral and axial resolutions remained largely unchanged with increasing frame rate; however, for concatenated B-scan images the lateral resolution decreased for high frame rates. Note that this is a result of the signal processing applied; the acoustic beam width is independent of the frame rate, but the greater velocity and corresponding sparser spatial sampling result in a less accurate estimation of the lateral resolution. The resolution and contrast values are summarized in Table 1.

**Table 1:** Summary of the imaging performance as measured using point targets. Values extracted from the data shown in Fig. 4. DaS, delay-and-sum.

	Concatenated B-scan			DaS-reconstructed		
Frame rate (Hz)	1	3	7	1	3	7
Axial resolution (μm)	100	94	112	192	192	168
Lateral resolution (μm)	440	440	600	281	241	281
Contrast (dB)	21	21	21	28	26	20

### 3.4 Phantom Imaging

**Fig. 5:**
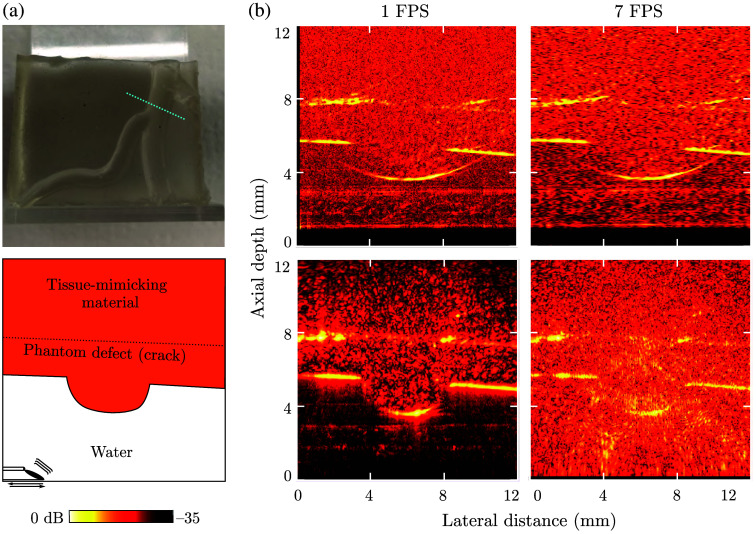
LiOpUS imaging of a tissue-mimicking phantom. (a) Photograph (top) of the phantom mimicking the vasculature found on the surface of a human placenta, with the imaging plane indicated by the dashed line. The imaging geometry and location of the LiOpUS probe are schematically depicted (bottom). (b) Corresponding LiOpUS images acquired at frame rates of 1 and 7 Hz, visualized as concatenated B-scans (top) and delay-and-sum reconstructed images (bottom). The location and translation direction of the LiOpUS probe are indicated in the bottom-left panel.

The LiOpUS platform achieved a strong contrast (up to 24 dB for DaS and 17 dB for concatenated B-scan images) of the phantom boundary, as well as a clear visualization of the subsurface defect and internal speckle pattern (Fig. 5). The concatenated B-scan imaging approach failed to fully recover the exterior structure of the phantom as the phantom boundary acted as a specular reflector, but the image contrast and quality remained largely unchanged with varying imaging frame rate. In contrast, DaS-reconstructed images—while still failing to fully visualize the phantom boundary—clearly visualized the full phantom geometry due to the recovered speckle pattern. However, the DaS-reconstructed images quickly degraded with increasing frame rate due to decreasing numbers of A-lines.

### 3.5 Interventional Imaging

**Fig. 6:**
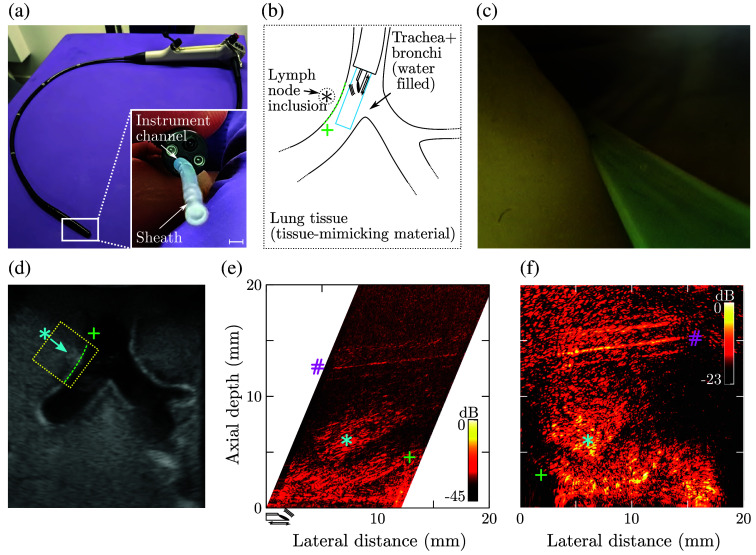
Interventional LiOpUS pulmonary phantom imaging. (a) LiOpUS imaging probe and its protective sheath were inserted into the instrument channel of a commercial bronchoscope. Scale bar: 2 mm. (b) This bronchoscope was used to position the LiOpUS probe in front of a hilar lymph node. (c) White light endoscopy image showing the phantom, sheath, and fiber-optic LiOpUS probe. (d) Ultrasound image of the phantom obtained using an electronic curvilinear imaging probe, showing the trachea, both the main stem bronchi, a hilar lymph node (*), and the LiOpUS imaging plane annotated in yellow. The bronchi continue throughout the phantom and merely appear truncated due to the orientation of the image plane. (e) and (f) LiOpUS images acquired at a frame rate of 1.0 FPS and displayed as concatenated B-scan (e) and DaS-reconstructed image (f) show the bronchial wall (+), lymph node (*), and an additional image artifact (#). The location and translation direction of the LiOpUS probe are indicated in panel (e). Videos of panels (c), (e), and (f) are provided in Video 1 (Video 1, MP4, 9.56 MB [URL: https://doi.org/10.1117/1.JBO.30.3.036005.s1]).

Emulated interventional imaging of an anatomically correct endobronchial phantom through a bronchoscope confirmed the compatibility of LiOpUS with current clinical instruments, as well as its capability for imaging in an interventional setting at clinically relevant image quality and at a frame rate of 1.0 Hz (Fig. 6). Video recordings of both the bronchoscope endoscopy view and the corresponding LiOpUS image can be viewed in Video 1. Both the bronchus and a subsurface lymph node structure were clearly visualized. An additional artifact can be observed at an axial depth of ca. 15 mm, which is likely a spurious reflection generated by additional photoacoustic coating material being deposited on the side of the fiber [cf. Fig. 2(e)]. This resulted in an additional acoustic emission orthogonal to the fiber long axis (in the upward direction in Fig. 2), which back-scattered off the opposite side of the bronchus.

## 4 Discussion and Conclusion

The LiOpUS imaging platform presented here achieved the first-ever sustained video-rate OpUS imaging in an interventional setting, using a miniature probe measuring just 600  μm laterally (1.8 mm including protective sheath). This platform achieved a high resolution (down to 241  μm lateral and 94  μm axial), dynamic range (up to 45 dB), an imaging depth of 20 mm, a field of view of ca. 15  mm, and a frame rate of up to 7 Hz—suitable for application in, for example, interventional endobronchial, cardiovascular, gastrointestinal, or neuroimaging procedures. Combined with a partially forward-facing field of view, the presented LiOpUS imaging probes enable image guidance during instrument insertion, a functionality not available in conventional interventional ultrasound probes of similar dimensions (e.g., IVUS or radial EBUS).

This work presented a method for fabricating obliquely emitting OpUS sources. This method, which uses a commercial laser cutter, is versatile, readily scalable, and reproducible, and hence shows great potential for future commercialization and clinical adaptation of LiOpUS. In this work, conformal coatings were deposited via simple dip-coating techniques, resulting in variable amounts of excess coating material being deposited on the side of the fibers (cf. Fig. 2) that generated additional spurious acoustic emissions (cf. Fig. 6). However, this direct dip-coating method could be replaced with a dip-transfer deposition approach to ensure only the generating surface is coated. Alternatively, the sides of the fibers could be prepared with a reflective layer (such as silver paint^9^) prior to laser cleaving and dip-coating to prevent acoustical emissions along the side of the fiber. Despite these unwanted additional emissions, an acoustic performance similar to previously reported work^12^^,^^22^ was achieved, resulting in an imaging depth of up to 20 mm—but further experiments are required to determine the maximum imaging depth of the system. In the experiments presented here with tissue-mimicking phantoms, the noise floor was lower than the signal from the tissue-mimicking material even at maximum imaging depth (cf. Fig. 6), suggesting that the LiOpUS system might be capable of deeper imaging still. The LiOpUS imaging performance (resolution: 241  μm lateral and 94  μm axial; frame rate: up to 7 Hz; field of view: 15 mm lateral by 20 mm axial; imaging plane oriented along probe long axis; probe diameter: 600  μm) compares favorably with those of radial EBUS (2 mm radial, 100  μm axial; ca. 20 Hz; up to 25 mm; cross-sectional; 1.4 to 2.6 mm)^32^ or IVUS (300  μm by 100  μm; ca. 20 Hz; 6 to 10 mm; cross-sectional; ca. 1 mm)^33^ imaging probes, albeit presently at lower frame rate.

Although the presented LiOpUS system was compatible with commercial interventional surgical tools, smaller protective sheaths might be used to further reduce the lateral size of the LiOpUS probe (potentially to a sub-millimeter scale) and expand the application area to include, for instance, robotic endobronchial or endovascular deployment. In this work, a fixed probe length of 1.5 m was used to allow deployment through the entire sheath length (ca. 1 m) and allow for versatile and deep future applications. Similar imaging performance is expected for shorter probe lengths; however, longer probes might experience increased friction that could limit motion accuracy. In addition, the third imaging scenario, that of the lung phantom, at present required full submersion of the phantom to ensure good acoustical coupling. However, future coupling could instead be achieved using inflatable balloons to accomplish physical contact between the sheath and tissue. Such balloons are commonly used during interventional procedures.

Presently, the imaging frame rate is limited primarily by the limited PRR of the excitation light source: for increasing frame rates, decreasing numbers of A-lines are recorded per frame. However, if a light source with higher PRR is used, higher-quality images at even higher frame rates are possible; in principle, the actuator is capable of periodic full-range back-and-forth motion at a frequency of 25 Hz. As imaging is performed in both the forward and backward directions, the maximum frame rate could be as high as 50 Hz. In this work, the aperture width was limited to 12 mm by the actuator, and although images can be reconstructed beyond this travel range (cf. Fig. 6), a wider aperture could be achieved by an actuator with a larger travel range. This would result in a wider field of view and, in the second image formation approach (DaS reconstructed), a higher lateral resolution. Combined with the oblique directivity of the LiOpUS imaging probe, a wider field of view would further enhance surgical navigation and interventional image guidance of, for example, biopsy needles or grabber tools, by offering an improved capacity to look ahead of the LiOpUS probe location. However, the field of view width of 12 mm already matches well with various clinically relevant targets, such as the lymph nodes, blood vessels, glands, and cardiovascular valves.

Currently, signal acquisition, processing, and display were performed at video rate and in real time. However, DaS reconstruction and SVD-based filtering could only be performed offline due to hardware restrictions in the experimental setup. These steps could be incorporated in real time using Graphical Processing Unit (GPU) processing. Efficient GPU-enabled beamformers have been presented^34^ that are easily capable of processing the amounts of data generated without impacting the imaging frame rate, and SVD-based filtering too could be offloaded to a GPU.

Further research is needed before clinical adaptation becomes possible, as the nature of the current approach to OpUS imaging presents some challenges. Delivering high-power and high-PRR excitation light onto a small photoacoustic coating occasionally resulted in microbubbles generated on the face of OpUS source causing a reduction in acoustic coupling, and the liquid flushing required to remove these bubbles might not be practical in a clinical setting. However, this could in future work be addressed through modification of the coating (for instance by making this more hydrophylic), coupling agent (e.g., using degassed water), or geometry (e.g. using a wider fiber to decrease fluence but increase directivity and hence penetration). In addition, the vigorous back-and-forth motion of the LiOpUS probe resulted in changes to the optimum bias wavelength of the fiber-optic sensor that somewhat reduced sensitivity, and additional research is required to address this limitation.

Despite current limitations and opportunities for improvement, the LiOpUS platform presented here already achieves sustained interventional imaging at clinically relevant image quality and frame rates. Its small form factor, potential low cost, and flexible design could significantly increase the quality and frequency of interventional ultrasound image guidance in a wide range of interventional scenarios, such as pulmonary, cardiovascular, gastrointestinal, or neuroimaging. In addition, a partially forward-facing field of view is expected to greatly improve interventional image guidance of instrument navigation and positioning.

## Supplementary Material

10.1117/1.JBO.30.3.036005.s1

## Data Availability

The code, data, and materials underlying the results presented in this paper are not publicly available at this time but may be obtained from the authors upon reasonable request.
